# Morphophysiological Adaptations of Aquatic Macrophytes in Wetland-Based Sewage Treatment Systems: Strategies for Resilience and Efficiency under Environmental Stress

**DOI:** 10.3390/plants13202870

**Published:** 2024-10-14

**Authors:** Leila Teresinha Maranho, Marcelo Pedrosa Gomes

**Affiliations:** 1 Plant Stress Physiology Laboratory, Department of Botany, Biological Sciences Sector, Polytechnic Center Jardim das Américas, Federal University of Paraná, Avenida Coronel Francisco H. dos Santos, 100, Curitiba 81531-980, Brazil; 2 Graduate Program in Ecology and Conservation, Federal University of Paraná, Curitiba 81531-980, Brazil

**Keywords:** contaminants, nature-based solution, phytoremediation, pollution, wastewater treatment

## Abstract

There is a common misconception that aquatic macrophytes face significant challenges in wetland-based sewage treatment systems. This study aims to correct this perception by focusing on the crucial morphophysiological adaptations of aquatic macrophytes that enable them to thrive in wetland-based sewage treatment systems, particularly under environmental stress. These adaptations are vital for improving the efficiency and resilience of wastewater treatment processes, offering sustainable solutions in the face of variable environmental conditions and complex contaminant mixtures. The review emphasizes the role of macrophytes as natural engineers, capable of enhancing pollutant removal and system stability through their unique structural and functional traits. By understanding these adaptations, the review aims to guide the optimization of wetland design and management, ultimately contributing to more sustainable and effective wastewater treatment practices. The findings underscore the importance of species selection and the integration of nature-based solutions in environmental management, advocating for policies that support the use of macrophytes in modern wastewater management.

## 1. Introduction

The need to address pressing environmental challenges has increased interest in nature’s capacity to adapt and flourish in adverse conditions [1]. Wetland-based sewage treatment systems have gained attention as a promising solution, harnessing the natural processes of ecosystems to treat wastewater sustainably and cost-effectively [2]. These systems stand out due to their ability to integrate ecological principles into modern wastewater management.

Aquatic macrophytes—large, aquatic plants that play a pivotal role in maintaining ecological balance—are central to the success of these wetland systems. These plants are not merely passive elements within the ecosystem; they actively contribute to nutrient cycling, water purification, and habitat formation [3]. Numerous studies underscore the ability of macrophytes to remove both organic and inorganic pollutants, significantly enhancing the efficiency of wetland-based sewage treatment systems [3,4,5,6,7]. However, applying macrophytes in sewage treatment environments is not without challenges. Under stressful conditions, such as high pollutant loads or adverse environmental factors, these plants may experience reduced growth rates and diminished nutrient removal efficiency [8]. For instance, research by Marques et al. [4] revealed that *Myriophyllum aquaticum*, despite being a strong candidate for phytoremediation, struggled to survive in wastewater effluents contaminated with antibiotics. The plant’s inability to tolerate these conditions, exacerbated by solid material accumulation on its leaves, ultimately hindered its photosynthetic capacity and led to its demise within three days of exposure. Despite these challenges, many aquatic macrophytes possess notable morphophysiological adaptations that allow them to survive, thrive, and efficiently remove pollutants [9,10], even under the stressful conditions commonly encountered in sewage treatment environments. These adaptations, including specialized root systems, efficient nutrient uptake mechanisms, and tolerance to low oxygen levels, are crucial for their resilience and effectiveness. Understanding these adaptive traits is critical for optimizing the use of macrophytes in wetland-based sewage treatment, ensuring that these systems remain efficient and resilient under varying environmental stresses.

This review examines the morphophysiological adaptations of aquatic macrophytes that enable them to survive and perform efficiently in wetland-based sewage treatment systems, even under environmental stress. By exploring these adaptations, we aim to illuminate how these plants can be utilized to optimize the design and maintenance of wetlands for wastewater treatment. Understanding these adaptive mechanisms offers crucial insights into enhancing the resilience and efficiency of sewage treatment processes, especially in the face of increasingly variable environmental conditions. While much of the existing research has focused on the phytoremediation capabilities of macrophytes, particularly their ability to remove specific contaminants, it is essential for considering the complex composition of wastewater treatment plant (WWTP) effluents. The presence of multiple contaminants and the additional stress imposed by climate change can significantly influence the efficacy of these plants, sometimes deviating from the results observed in controlled laboratory settings. However, by delving into the morphophysiological mechanisms of each macrophyte morphotype, we can better harness their potential to optimize treatment systems, even in the face of environmental challenges. This approach positions macrophytes as natural engineers capable of enhancing wastewater treatment processes under various conditions. The novelty of this review lies in its dual focus: it highlights the unique adaptations of macrophytes with their practical applications in improving sewage treatment methods. This allowed us to understand aquatic macrophytes’ role in wetland-based sewage treatment systems and their broader implications for environmental management.

## 2. Aquatic Macrophytes in the Treatment System

Aquatic macrophytes have transitioned from terrestrial to aquatic environments, evolving specific morphophysiological adaptations essential for their survival and functionality in water. These adaptations include structural, morphological, and anatomical changes, such as a thin cuticle to minimize water loss, aerenchyma for oxygen storage, hollow stems to facilitate flotation, and flowers extending above the water surface to aid reproduction. This diverse group of plants includes angiosperms, ferns, mosses, liverworts, and green algae, which can thrive in various aquatic settings—from the margins of water bodies to fully submerged or floating on the surface [11,12,13].

Aquatic macrophytes are integral to wetland-based sewage treatment systems, because they absorb and remove pollutants from wastewater [4]. These macrophytes can be classified into four distinct morphotypes: emergent, submerged, floating-leaved, and free-floating [3]. Each of these morphotypes possesses unique physical and ecological characteristics that make them well-suited for specific functions in wastewater treatment (Table 1).

Emergent macrophytes are characterized by their growth with roots anchored in the sediment, while their stems and leaves extend above the water’s surface. Common examples include cattails (*Typha* spp.), bulrushes (*Schoenoplectus* spp.), and reeds (*Phragmites* spp.). These plants are precious in wastewater treatment systems due to their rapid growth and high capacity for pollutant removal [14,15]. They are well-suited to the fluctuating water levels typical of wetlands and effectively remove nutrients and organic matter from wastewater [16]. Most emergent macrophytes are amphibious, meaning they can thrive in waterlogged conditions and on land, making them versatile for various wastewater treatment systems (Table 1).

Floating-leaved macrophytes, including water lilies (*Nymphaea* spp.) and lotus (*Nelumbo* spp.), have roots anchored in the sediment, but their leaves float on the water’s surface. These plants contribute to wastewater treatment by reducing light penetration, suppressing algal growth, and absorbing nutrients through their extensive root systems [9,15,17]. The shading provided by their leaves also helps moderate water temperatures, creating a more stable environment for other aquatic organisms and enhancing overall water quality. Free-floating macrophytes, such as duckweed (*Lemna* spp.), water hyacinth (*Eichhornia crassipes*), and water lettuce (*Pistia stratiotes*), are not anchored to the substrate and float freely on the water’s surface. These plants are particularly effective in the initial stages of wastewater treatment due to their rapid growth and high nutrient uptake capacity. Free-floating macrophytes quickly reduce light penetration by covering large surface areas, inhibiting algal growth. Their roots also provide habitats for microorganisms that degrade organic pollutants (Table 1). However, their rapid growth presents challenges, as they can become invasive if not carefully managed [18].

Submerged macrophytes are aquatic plants that grow entirely underwater, with only their flowers sometimes reaching the surface. Examples include pondweeds (*Potamogeton* spp.), watermilfoils (*Myriophyllum* spp.), and hornworts (*Ceratophyllum* spp.). These plants are crucial in aquatic ecosystems, particularly wetland-based sewage treatment systems. Their primary functions include oxygenating the water column, essential for supporting aerobic microorganisms that break down organic pollutants. Submerged macrophytes absorb nutrients directly from the water, helping to prevent eutrophication—a process where excessive nutrients lead to harmful algal blooms. By competing effectively with algae for light and nutrients, these plants help maintain water clarity and quality. Additionally, submerged macrophytes provide habitat and food for various aquatic organisms, further contributing to the ecological balance of wetland environments. Their presence in wastewater treatment systems enhances nutrient removal efficiency, making them a valuable component of these systems [19,20].

The removal efficiency of contaminants in wetland-based sewage treatment systems can vary significantly among different types of aquatic macrophytes, mainly due to their differing abilities to acclimate to the effluents of WWTPs. For instance, submerged macrophytes, such as *M. aquaticum* and *Rotala rotundifolia*, have been observed to remove more of the antibiotic erythromycin from water (31 to 44% removal efficiency) than the floating species *Salvinia molesta* and *L. minor* (9 to 12% removal, respectively) [20]. This difference is attributed to several factors, including anatomical and morphological characteristics. The epidermises of submerged plants are particularly effective at adsorbing antibiotics, which enhances their contaminant removal capacity. Moreover, submerged plants often exhibit higher antibiotic metabolism rates than floating species, further contributing to their superior ability to remove erythromycin from aquatic environments. Although this phenomenon warrants further study, current evidence suggests that submerged aquatic macrophytes are generally more efficient at eliminating erythromycin from water than their floating counterparts [20].

Careful selection of macrophyte species, considering their geographical distribution, growth patterns, and maintenance needs (Table 2), can optimize the performance of water and sewage treatment systems. Species such as *Chrysopogon zizanioides* and *Thalia geniculata* have minimal maintenance needs. They effectively remove nutrients and organic matter, making them valuable for treatment systems where efficiency and ease of management are essential [21,22]. Other species, such as *Cyperus papyrus* and *Phalaris arundinacea*, also demonstrate effectiveness with low maintenance requirements [23,24]. The flexibility of macrophytes in terms of cultivation methods and treatment conditions underscores their importance in optimizing wastewater treatment processes and aligning species selection with the specific needs of each project.

Another crucial aspect to consider when selecting macrophytes for treating effluents from WWTPs is the water quality, as different macrophyte species have varying sensitivities to environmental conditions. Submerged macrophytes, for instance, are particularly sensitive to oxygen availability and light penetration. High concentrations of dissolved solids, which affect water color and turbidity, can constrain the use of these species by reducing light availability and oxygen levels, both of which are essential for their survival and efficiency in contaminant removal [4,25]. In contrast, emergent macrophytes are more resilient to these challenges. They survive in low-oxygen environments and contribute to water quality by injecting oxygen into the water through specialized tissues known as aerenchyma [26]. This oxygenation can benefit other aquatic organisms and enhance overall treatment efficiency. While effective in nutrient uptake, floating macrophytes can be sensitive to pH variations [27]. Extremes in pH levels can negatively impact their growth and survival, making them less suitable in environments where the water chemistry is highly variable. Therefore, carefully considering water quality parameters, such as oxygen concentration, turbidity, and pH, is essential for selecting the most appropriate macrophyte species for effective and sustainable wastewater treatment.

In summary, aquatic macrophytes are essential for wetland-based sewage treatment systems, providing various biological and ecological benefits. Their diverse morphotypes and adaptations enable them to perform multiple roles in pollutant removal, habitat stabilization, and water quality enhancement. By carefully selecting and managing these plants, it is possible to optimize the efficiency and resilience of wastewater treatment systems, ensuring they meet the specific environmental and operational needs of different projects.

**Table 2:** Main macrophyte species used in treatment systems. Summary of their geographical distribution, growth and reproduction characteristics, and maintenance requirements.

Macrophyte Species	Geographical Distribution	Growth and Reproduction	Required Maintenance	Source
*Acorus calamus*	Tropical and subtropical	Moderate, reproduces by rhizomes and seeds	Low	Hua et al. [23]
*Arundo donax*	Mediterranean and subtropical	Rapid, reproduces by rhizomes	Moderate	Du and Song [28]
*Canna indica*	Tropical and subtropical	Rapid, reproduces by rhizomes and seeds	Low	Yadav et al. [29]
*Canna x generalis*	Tropical and subtropical	Rapid, reproduces by rhizomes and seeds	Low	Chaves et al. [30]
*Chrysopogon zizanioides*	Tropical and subtropical	Rapid, reproduces by rhizomes	Minimal	Waqkene et al. [21]
*Coix lacryma-jobi*	Tropical and subtropical	Moderate, reproduces by seeds	Minimal	Chaves et al. [30]
*Cyperus alternifolius*	Tropical and subtropical	Rapid, reproduces by rhizomes	Minimal	Corzo and Sanabria [24]
*Cyperus articulatus*	Tropical and subtropical	Moderate, reproduces by rhizomes	Low	Caselles-Osorio et al. [31]
*Cyperus haspan*	Tropical and subtropical	Rapid, reproduces by rhizomes	Minimal	Henry et al. [32]
*Cyperus papyrus*	Tropical and subtropical	Rapid, reproduces by rhizomes	Low	García-Ávila et al. [33]
*Dioscorea* spp.	Tropical and subtropical	Rapid, reproduces by rhizomes	Minimal	Chaves et al. [30]
*Eichhornia crassipes*	Global	Rapid reproduction by fragmentation	High	Kumari et al. [34]
*Erianthus arundinaceus*	Tropical and subtropical	Rapid, reproduces by rhizomes	Minimal	Arivoli et al. [35]
*Heliconia burleana*	Tropical and subtropical	Rapid, reproduces by rhizomes	Minimal	Corzo and Sanabria [24]
*Heliconia zingiberales*	Tropical and subtropical	Rapid, reproduces by rhizomes and seeds	Minimal	Trejo-Téllez [36]
*Imperata cylindrica*	Tropical and subtropical	Rapid, reproduces by rhizomes	Minimal	Khajah and Ahmed [37]
*Iris pseudacorus*	Europe, Asia, and North America	Moderate, reproduces by rhizomes and seeds	Low	Huang et al. [38]; Yao et al. [39]
*Juncus acutus*	North and Central America	Moderate, reproduces by rhizomes and seeds	Minimal	Zahran et al. [40]
*Leptochloa fusca*	Tropical and subtropical	Rapid, reproduces by rhizomes	Minimal	Snow et al. [41]
*Lolium multiflorum*	Temperate	Rapid, reproduces by seeds	Moderate	Vila-Aiub et al. [42]
*Melaleuca quinquenervia*	Australia and Pacific	Rapid, reproduces by seeds and rhizomes	Moderate	Bolton et al. [43]
*Phalaris arundinacea*	Temperate and subtropical	Rapid, reproduces by rhizomes	Low	Sochacki et al. [44]
*Phragmites australis*	Global	Rapid, reproduces by rhizomes	Low	Hussain et al. [45]; Malyan et al. [46]; Jain et al. [47]; Arivoli et al. [35]
*Phragmites* spp.	Global	Rapid, reproduces by rhizomes	Low	Redder et al. [48]
*Pontederia cordata*	North and Central America	Rapid, reproduces by seeds and rhizomes	Low	Chang et al. [49]
*Scirpus alternifolius*	Tropical and subtropical	Rapid, reproduces by rhizomes	Low	Villar et al. [50]
*Scirpus grossus*	Tropical and subtropical	Rapid, reproduces by rhizomes	Low	Sun et al. [51]
*Thalia geniculata*	Tropical and subtropical	Rapid, reproduces by seeds and rhizomes	Minimal	Obeng et al. [22]
*Typha angustata*	Asia and Africa	Rapid, reproduces by rhizomes	Low	Nguru and Sabo [52]
*Typha angustifolia*	Tropical and subtropical	Rapid, reproduces by rhizomes	Low	Malyan et al. [46]; Arivoli et al. [35]; Arliyani et al. [53]
*Typha latifolia*	Global	Rapid, reproduces by rhizomes	Minimal	Malyan et al. [46]
*Vetiveria zizanioides*	Tropical and subtropical	Rapid, reproduces by rhizomes	Minimal	Almeida et al. [54]
*Zantedeschia aethiopica*	Tropical and subtropical	Rapid, reproduces by seeds and rhizomes	Minimal	Corzo and Sanabria [24]
*Zingiber officinale*	Tropical and subtropical	Rapid, reproduces by rhizomes	Minimal	Chaves et al. [30]

### 2.1. Function of Macrophytes in Treatment Systems

**Figure 1:**
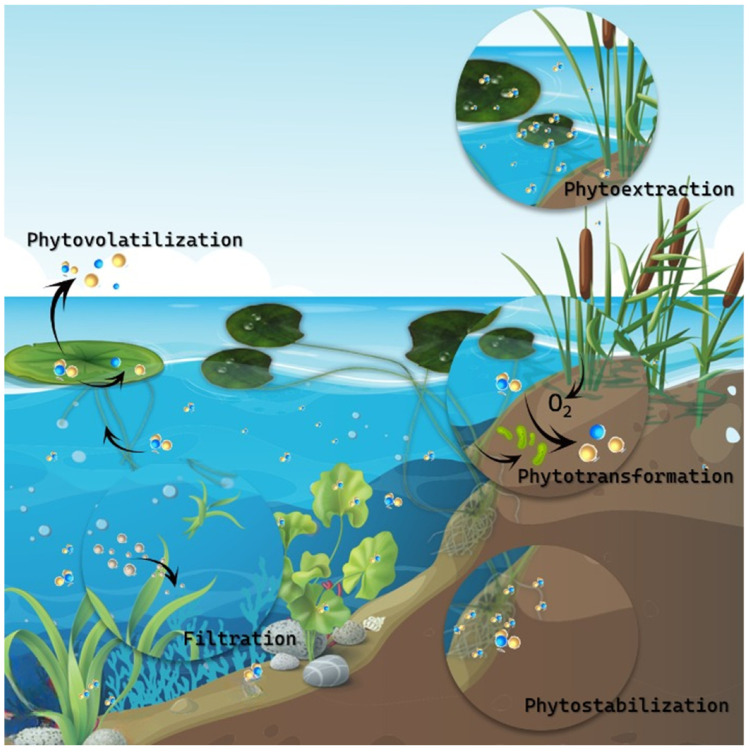
Phytoremediation strategies employed by aquatic macrophytes in wetland-based sewage treatment systems. Phytovolatilization occurs as plants absorb contaminants and release them as gases through transpiration. Phytoextraction involves plants absorbing pollutants from the water and/or sediment, accumulating them in their tissues. Phytotransformation highlights the chemical transformation of contaminants through plant or microorganism metabolism. Phytostabilization is depicted in the root zone of emergent plants, where contaminants are immobilized in the sediment, reducing their mobility and environmental impact. Additionally, macrophytes capture particles and enhance sediment stability, decreasing turbidity and clearing the water. They also improve microbial activity within the treatment system, with some microorganisms collaborating in contaminant metabolism.

Aquatic macrophytes play a critical role in wetland-based sewage treatment systems, offering a range of benefits that enhance the removal of pollutants and improve overall treatment efficiency. Their unique morphophysiological adaptations enable them to perform vital functions, such as nutrient removal, organic pollutant degradation, heavy metal phytoextraction and phytotransformation, sediment filtration, and enhancing system efficiency through promoting microbial activity (Figure 1).

#### 2.1.1. Nutrient Removal

Nutrient removal is one of the primary functions of macrophytes in wastewater treatment. These plants are highly effective in removing nutrients, such as nitrogen and phosphorus, commonly found in wastewater, and can lead to eutrophication if not correctly managed. The removal of nitrogen involves both nitrification and denitrification processes, where bacteria in the root zone of macrophytes convert ammonia to nitrate and then to nitrogen gas, which is released into the atmosphere. On the other hand, phosphorus is either assimilated by the plants or precipitated with mineral compounds in the sediments [39] (Table 3). Macrophytes, such as *Typha* spp. and *Phragmites* spp., are particularly efficient at nutrient removal due to their ability to absorb and accumulate these elements in their biomass [47,55]. Studies have shown that in just seven days of exposure, *L. gibba* can remove nearly 50% of phosphate from WWTP effluents [4]. Additionally, macrophytes enhance microbial activity in the root zone, which plays a crucial role in the degradation of nutrients, thereby improving the overall efficiency of the treatment system [56].

#### 2.1.2. Organic Pollutant Removal

In addition to nutrient removal, aquatic macrophytes employ several phytoremediation strategies to remove organic pollutants, such as phenolics, pharmaceuticals, and other complex compounds (Figure 1). Phytostabilization immobilizes contaminants within the root zone, reducing their mobility and environmental impact. Floating species such as *Lemna* sp. and *Azolla* sp. effectively utilize phytostabilization to remove pharmaceuticals and personal care products [47,48]. Phytovolatilization allows plants to absorb pollutants and release them as gases through transpiration. This strategy is particularly effective for volatile organic compounds with low octanol-air partitioning coefficients [57]. Macrophytes have been shown to volatilize various organic contaminants, including selenium, ethylene dibromide, and carbon tetrachloride [57]. However, incomplete degradation during this process can sometimes lead to phytotoxicity and additional environmental concerns [58]. Genetic engineering techniques can improve plants’ tolerance, accumulation, and detoxification capabilities to enhance efficiency and reduce risks.

Phytoimmobilization involves the sequestration of contaminants within plant tissues, particularly in the roots, which prevents their spread through the ecosystem. Floating macrophytes are especially effective in immobilizing organic pollutants, such as pharmaceuticals [4,59,60] and pesticides [61,62]. Recent studies by Marques et al. [4] have demonstrated the phytoimmobilization capacity of macrophytes, such as *S. minima*, *Sagittaria montevidensis*, and *L. gibba*, in removing various antibiotics from WWTP effluents. These antibiotics include sulfamethoxazole, sulfadiazine, four quinolones (ciprofloxacin, enrofloxacin, norfloxacin, and levofloxacin), three tetracyclines (oxytetracycline, tetracycline, doxycycline), one macrolide (azithromycin), and two β-lactams (amoxicillin, meropenem). After just seven days of exposure, these plants removed between 12% and 80% of the antibiotics, demonstrating the efficiency of using macrophytes in post-treatment processes for WWTP effluents.

**Table 3:** Mechanisms of nutrient uptake and accumulation by aquatic macrophytes.

Mechanism	Description	Key Structures/Processes	References
Root Uptake	Nutrients are absorbed from the soil or sediment through root systems	- Root hairs: increase surface area for nutrient absorption. - Mycorrhizal associations: enhance nutrient uptake and efficiency.	Palaicos et al. [63] Kalengo et al. [64]
Nutrient Assimilation	Absorbed nutrients are incorporated into plant biomass or stored in vacuoles	- Nitrogen: incorporated into amino acids, proteins, and nucleic acids. - Phosphorus: integrated into nucleic acids, ATP, and phospholipids.	Reddy and DeLaune [48]; Beilby et al. [65]
Storage Mechanisms	Nutrients are sequestered in various plant tissues to prevent their release into the water column.	- Phosphorus storage: often stored in vacuoles within roots and stems. - Nitrogen: stored as organic compounds within plant tissues.	Vymazal [66]; Nikilakipoulou et al. [67]

In phytodegradation, plants and associated microbial communities metabolize organic compounds through processes similar to human drug metabolism, including oxidation, reduction, and conjugation [68]. Submerged macrophytes are particularly effective in this process due to their ability to oxygenate the water, which enhances microbial activity essential for further pollutant breakdown. However, a potential concern with phytodegradation is that the by-products generated can sometimes be more toxic than the original pollutants, posing challenges for using macrophytes in post-treatment systems.

#### 2.1.3. Heavy Metals Removal

Heavy metal removal is a critical function of macrophytes in wastewater treatment systems, achieved through phytoimmobilization, phytostabilization, phytoextraction, and phytotransformation (Figure 1). Phytoimmobilization involves the absorption and sequestration of heavy metals within the plant roots, effectively trapping the contaminants and preventing them from entering the food chain or spreading through the environment. In contrast, phytostabilization keeps contaminants immobilized in the sediment, reducing their bioavailability and, thus, minimizing their environmental impact by preventing uptake by other organisms.

Phytoextraction involves macrophytes absorbing dissolved heavy metals from water or soil through their roots and translocating them to their stems and leaves, where they accumulate. Over time, the accrued metals can be removed from the environment by harvesting the plants. Various species, including *E. crassipes*, *P. stratiotes*, and *L. minor*, have demonstrated high efficiency in extracting metals such as chromium, copper, nickel, lead, cadmium, and iron from water [69,70]. The capacity for metal accumulation varies among species and plant tissues, with roots generally accumulating higher concentrations than shoots [71]. Factors affecting heavy metal removal efficiency include plant life form, biomass, age, metal type, initial concentration, and water chemistry. While the uptake of metals by macrophytes offers a cost-effective and eco-friendly solution for metal pollution abatement, challenges, such as biomass disposal and seasonal growth limitations, remain [72,73].

In addition to these methods, macrophytes can also engage in phytotransformation. In this process, plants alter the chemical form of heavy metals within their tissues through enzymatic reactions, converting toxic metals into less harmful or more stable forms [74]. This process reduces metal bioavailability and environmental impact. Phytotransformation typically involves three stages: bioactivation, conjugation, and compartmentalization, each requiring specific enzymes, such as oxygenases and nitroreductases, which are classified based on the properties and distribution of their reaction products [74]. For example, *P. australis* and *T. latifolia* transform heavy metals, such as zinc, copper, mercury, and lead into less toxic forms, which can then be sequestered in specific plant parts [75]. Macrophytes that engage in phytostabilization often act as excluders, limiting the translocation of metals within the plant and maintaining bioaccumulation factors generally below [76]. It is important to note that the phytoremediation strategy used by a macrophyte species can vary depending on the specific contaminant, with species such as *Typha* spp. and *Phragmites* spp. particularly effective in phytostabilization in constructed wetlands [77].

Another technique macrophytes use is rhizofiltration, which utilizes plant roots to absorb, concentrate, and precipitate toxic metals from aqueous environments [78]. This technique is particularly effective for removing heavy metals but can also be applied to organic contaminants [79]. Contaminants either accumulate within the root tissues or precipitate on the root surfaces, where they can be removed when the plants are harvested [78]. Additionally, these roots can alter the pH and redox conditions in the surrounding soil, further reducing the solubility of heavy metals and other pollutants. Rhizofiltration involves complex plant–microbe interactions in the rhizosphere, where root-associated bacteria play a crucial role in degrading organic pollutants and transforming inorganic contaminants [80]. While rhizofiltration shows promise for large-scale applications, further research is needed to enhance its efficiency and overcome limitations, potentially through genetic engineering and biotechnological approaches [79].

#### 2.1.4. Oxygenation

Particularly in the case of submerged and emergent macrophytes, macrophytes contribute to the oxygenation of the water column through radial oxygen loss from their roots, creating oxidized microenvironments that stimulate carbon and nitrogen transformations [81,82]. These plants play a crucial role in oxygenating the water column. Submerged macrophytes release oxygen through photosynthesis, essential for the survival of aerobic microorganisms that decompose organic matter in the water. On the other hand, emergent macrophytes contribute to oxygen levels by transporting oxygen from the atmosphere to their roots through specialized tissues known as aerenchyma, which also helps improve conditions in the rhizosphere, the root-affected zone in the sediment. This process is particularly effective in emergent species, such as *T. domingensis*, and floating plants, such as *E. crassipes* [83].

#### 2.1.5. Stabilization of Sediments

Macrophytes play a vital role in stabilizing sediments in wetland environments, preventing erosion, and reducing the resuspension of pollutants. Their extensive root systems anchor the soil, minimizing sediment movement, which is crucial for maintaining water clarity and preventing the spread of contaminants. This stabilization supports the ecosystem’s overall health and enhances the efficiency of submerged plants that require clear water for photosynthesis. Both emergent and submerged species are highly effective in decreasing sediment resuspension and improving water quality [84,85]. Species such as *Echinochloa stagnina* and *T. angustifolia* are particularly effective at trapping sediments and preventing their resuspension, thus contributing to more transparent water and a more stable aquatic environment [86]. Recent studies have demonstrated that macrophytes can improve water turbidity by intercepting micro- and nanoplastics, further highlighting their role in maintaining water quality [87].

#### 2.1.6. Habitat Provision

Aquatic macrophytes provide critical habitats for aquatic organisms, including invertebrates, fish, and microorganisms [10]. These habitats support biodiversity within the treatment system, which can enhance the breakdown of organic matter and the cycling of nutrients [10]. The complex structure of macrophytes, particularly submerged and floating-leaved types, offers shelter and breeding grounds for these organisms, which in turn contribute to the overall functioning of the ecosystem.

#### 2.1.7. Light and Temperature Regulation

Floating-leaved and free-floating macrophytes are crucial in regulating light penetration in aquatic environments. By covering the water surface, these plants reduce the amount of light reaching submerged layers, which helps control algal growth [88]. Excessive nutrient loads can lead to algal blooms that deplete oxygen levels and hinder the overall efficiency of wastewater treatment systems. By limiting light availability, macrophytes maintain a balanced light environment that supports the health of the aquatic ecosystem. Additionally, macrophytes can suppress algal growth through nutrient competition and the release of allelopathic chemicals, which inhibit algal development [19]. However, these allelopathic effects do not impact all photosynthetic organisms equally. Recent studies by Kochi et al. [5] observed a possible positive allelopathic interaction between *L. minor* and *S. molesta*. When co-cultured and exposed to the antibiotics ciprofloxacin (1.5 µg L^−1^) and sulfamethoxazole (0.3 µg L^−1^), *L. minor* and *S. molesta* exhibited increased growth rates, leading to more excellent antibiotic removal in mixed systems compared to monocultures.

The shading effect of floating-leaved macrophytes also helps moderate water temperatures, creating a more stable environment for other aquatic organisms and processes. This temperature regulation is essential for preventing the overheating of water bodies, which can negatively impact both biological activity and the physical properties of the water. The authors of [4] observed a decrease of about 1 °C in effluents from WWTPs after seven days of treatment with *L. minor*, compared to the temperature of systems without plants. In the context of climate change, reducing the temperature of effluents before they enter rivers may help mitigate the potential harmful effects of increasing water temperatures, contributing to the resilience of aquatic ecosystems.

#### 2.1.8. Enhancement of Microbial Activity

The presence of macrophytes significantly enhances microbial activity within wetland-based treatment systems. Macrophytes provide extensive surfaces for microbial attachment and release root exudates that can alter microbial diversity and composition [56]. Additionally, these plants create aerobic zones by releasing oxygen from their roots, essential for supporting aerobic microorganisms that play a crucial role in nutrient cycling and the decomposition of organic matter. Macrophytes have been shown to increase microbial density, enzymatic activity, and metabolic functions within constructed wetlands [87]. The interaction between macrophytes and these microbial communities is vital for the biogeochemical cycling of nutrients and the overall efficiency of the wastewater treatment process [89]. This symbiotic relationship not only enhances the breakdown of contaminants but also improves the resilience and sustainability of the treatment system.

#### 2.1.9. Filtration Sediment Reduction

Macrophytes are crucial in filtration and sediment reduction within wetland-based sewage treatment systems [89,90]. Their root structures act as natural filters, trapping suspended solids and preventing the resuspension of sediments, which helps maintain water clarity. This filtration process is essential for reducing the load of particulate matter, including organic and inorganic contaminants, from the water column. Macrophytes such as *S. lacustris* and *E. stagnina* are especially effective at trapping sediments and preventing resuspension [67,86]. With their extensive and robust root systems, emergent macrophytes excel at stabilizing sediments and reducing erosion. By anchoring the soil, these plants minimize sediment movement, which is essential for preventing the spread of attached pollutants and ensuring a stable aquatic environment. Submerged and floating macrophytes also contribute to sediment stabilization by capturing fine particles that might remain suspended in the water [91].

The reduction in sediment resuspension by macrophytes not only improves water quality but also enhances the overall efficiency of the treatment system. More transparent water allows more light to penetrate, benefiting photosynthetic organisms and maintaining the ecological balance within the wetland. Additionally, stabilizing sediments by macrophytes reduces turbidity and prevents the release of previously settled contaminants, further protecting the aquatic environment.

## 3. Morphophysiological Adaptations of Aquatic Macrophytes to Sewage Treatment Systems

Aquatic macrophytes have evolved a range of morphophysiological adaptations that enable them to thrive in the challenging conditions of sewage treatment systems. These adaptations are crucial for their survival and functionality, allowing them to efficiently remove pollutants, stabilize sediments, and support microbial activity in wetland environments. This section explores the key adaptations that make aquatic macrophytes well-suited to their roles in sewage treatment systems.

### 3.1. Structural Adaptations

One of the most significant adaptations of aquatic macrophytes is the development of specialized root systems capable of anchoring the plants in soft, often unstable sediments, while facilitating the uptake of nutrients and contaminants. Emergent macrophytes, such as *Typha*, *Eleocharis*, and *Phragmites* species, possess extensive and robust root networks that stabilize sediments and extend deep into the substrate, accessing nutrients and pollutants from a wide area [67,86]. These roots are often equipped with aerenchyma—spongy tissues that allow for the diffusion of oxygen from the aerial parts of the plant down to the roots [26]. Oxygen movement occurs through diffusion and pressurized convection in some species [26]. This adaptation is critical in waterlogged environments where oxygen is limited, enabling the roots to perform essential metabolic processes even in anaerobic conditions [92].

### 3.2. Physiological Adaptations

Aquatic macrophytes exhibit several physiological adaptations that significantly enhance their ability to process and remove contaminants from water in sewage treatment systems, including nutrients and metals. One of the critical physiological adaptations is the presence of aerenchyma, specialized tissues that facilitate oxygen transport within the plant and support radial oxygen loss (ROL). This process creates oxidized microzones around the roots [93], crucial for nitrification, where ammonia is converted to nitrate, and for oxidizing other pollutants. These oxidized microzones enhance the plant’s ability to absorb nutrients and metals or support their degradation by associated microbial communities. Many wetland plants also form barriers to ROL in the basal parts of their roots, enhancing oxygen diffusion to the root tips, while impeding the entry of phytotoxins [94]. These barriers, mainly composed of suberin, can develop in both adventitious and lateral roots [95]. ROL patterns vary across different spatial and temporal scales, influencing rhizosphere oxygenation and the structure of microbial communities [96]. While some plants lack these ROL barriers, they compensate by modifying their root architecture to optimize oxygen dynamics [97].

Regarding nutrient uptake, macrophytes have evolved efficient strategies to maximize the absorption and utilization of nutrients, which also contributes to the reduction in metals in sewage treatment systems. Many macrophytes exhibit high nutrient uptake capacities, absorbing nitrogen and phosphorus through their extensive root systems [3]. These nutrients are either assimilated into plant biomass or precipitated as mineral compounds in the sediment, reducing their availability in the water column. The ability to accumulate nutrients in plant tissues also aids in the phytoextraction of metals, as these nutrients often bind to metal ions, facilitating their uptake and removal from the water. For instance, the internal phosphorus, nitrogen, and sulfur concentrations have been linked to plants’ tolerance and metal remediation capacity [98]. Therefore, a high nutrient uptake rate supports plant survival and enhances phytoremediation capacity. Macrophytes can also mitigate the phytotoxic effects of metals by reducing their translocation to aerial parts of the plants or by transforming harmful substances into less toxic forms [72]. Additionally, the release of root exudates, which include organic acids and enzymes, can alter the pH and redox conditions in the rhizosphere, increasing the solubility and availability of nutrients and metals for uptake [56].

Macrophytes have also adapted to tolerate high levels of pollutants, including heavy metals and organic contaminants. This tolerance is often mediated by the production of specific enzymes and proteins that detoxify harmful substances or classify them within plant tissues, reducing their impact on vital physiological processes. Stressful conditions, such as the presence of multiple contaminants, can lead to the overproduction of reactive oxygen species (ROS), which can compromise the performance of plants in phytoremediation. However, macrophytes are well-adapted to such conditions. They increase the activity of antioxidant enzymes, such as superoxide dismutase, and hydrogen peroxide-scavenging enzymes, such as ascorbate peroxidase and catalase, enabling them to tolerate and continue growing, while removing various contaminants, including metals and pharmaceuticals, from water [3,5,99,100,101]. The central role of these antioxidant enzymes in the phytoremediation capacity of *L. minor* was confirmed by using specific inhibitors of their activities [102].

In addition to these detoxification mechanisms, aquatic macrophytes have evolved to optimize photosynthesis under the specific conditions found in sewage treatment systems. Most aquatic macrophytes utilize the C_3_ photosynthetic pathway, which is well-suited to the stable, water-abundant environments these plants typically inhabit [103]. However, they have also developed physiological adaptations that enable efficient carbon fixation and survival under varying light intensity conditions and water availability. These adaptations include light stress tolerance or avoidance strategies, such as investment in leaf area, adjustments in photosynthetic efficiency (including the strategic arrangement of chloroplasts and regulation of stomatal conductance), and vertical growth to optimize light capture [103,104,105]. Further adaptations include adjustmen3ts in light-harvesting capacity, such as changes in chlorophyll concentrations and modifications in carbon and nitrogen metabolism, which allow for more efficient energy use under different light conditions. Additionally, macrophytes demonstrate flexibility in biomass allocation and nutrient uptake, allowing them to allocate resources effectively depending on the environmental conditions [106,107]. These combined adaptations enable macrophytes to maintain high photosynthesis and growth rates in nutrient-rich environments, making them highly effective in sewage treatment applications.

### 3.3. Morphological and Anatomical Adaptations

Aquatic macrophytes display a range of structural and morphological adaptations that enable them to thrive in the demanding environments of sewage treatment wetlands. These adaptations are crucial for their survival and improving water treatment efficiency. For instance, many macrophytes’ large leaf surfaces and flexible stems allow these plants to maximize light capture for photosynthesis, while withstanding the physical stresses imposed by moving water and fluctuating water levels. This flexibility reduces mechanical damage and helps these plants maintain stability in dynamic hydrological conditions, which is essential for continuous treatment performance [108].

Floating-leaved species, such as *Nymphaea* spp., possess specialized air-filled aerenchyma tissues that provide buoyancy, keeping their leaves at the water’s surface. This structural adaptation ensures that the plants can capture maximum sunlight for photosynthesis, a critical process in nutrient-rich wetlands, where efficient photosynthesis directly enhances nutrient uptake and overall plant productivity. Additionally, the shading effect created by these floating leaves helps regulate water temperature and light penetration, which is beneficial for controlling algal growth and maintaining ecological balance within the treatment system [108]. Submerged macrophytes, such as *Elodea canadensis* and *Vallisneria* spp., often have finely divided leaves that increase the surface area available for gas exchange and nutrient absorption. This morphological trait is vital for maintaining high rates of photosynthesis and nutrient uptake in environments, where nutrient levels can vary significantly. These finely divided leaves allow submerged macrophytes to efficiently uptake dissolved gases and nutrients, enabling them to thrive even in low-light conditions often found in densely vegetated wetland areas [109]. Emergent species, such as *Typha* spp., *Phragmites* spp., and *Carex* spp., are equipped with robust, erect stems that allow them to grow through layers of sediment and maintain contact with the atmosphere. This adaptation is essential for sustaining gas exchange and nutrient uptake in wetland environments, where the sediment is often highly anoxic. The ability of these plants to extend their stems above the water surface facilitates oxygen exchange, preventing the buildup of anaerobic conditions in the root zone, which is crucial for promoting aerobic microbial processes vital for nutrient cycling and contaminant degradation [110].

The root systems of many aquatic macrophytes are also specially adapted to enhance their role in sediment stabilization and nutrient absorption. For example, *T. latifolia* has a dense, fibrous root system that anchors the plant in place, while stabilizing sediments and reducing erosion. This not only supports the plant’s structural integrity but also plays a significant role in trapping contaminants and preventing nutrient loss, thus contributing to the overall health and efficiency of the wetland treatment system. Additionally, these roots often feature increased root hair density, which expands the surface area for nutrient and contaminant absorption. The secretion of organic acids and enzymes by roots can also modify pH and redox conditions in the rhizosphere, increasing the availability of nutrients and metals for uptake and further enhancing the plant’s phytoremediation capacity [56].

Aquatic macrophytes have evolved to optimize photosynthesis under specific conditions in sewage treatment systems, such as light intensities and water availability variations. These adaptations include investment in leaf area to increase light harvesting and adjustments in stomata distribution and density to optimize gas exchange under fluctuating conditions [103,104]. Moreover, many macrophytes develop large central vacuoles that store harmful substances, such as heavy metals, thus reducing their toxicity and enabling the plant to thrive in contaminated environments. Thickened cell walls in these plants, often rich in lignin and suberin, further contribute to their resilience by binding and immobilizing contaminants, reducing their bioavailability, and preventing their translocation within the plant [111]. Combining these structural and morphological adaptations enables aquatic macrophytes to survive and thrive in the challenging conditions of sewage treatment wetlands. By improving nutrient cycling, enhancing water quality, and stabilizing sediments, these plants play a vital role in the sustainability and resilience of wastewater treatment systems. Understanding these adaptations optimizes wetland design and management, ensuring these systems operate efficiently and sustainably under various environmental conditions.

### 3.4. Reproductive Strategies and Population Dynamics

Aquatic macrophytes exhibit various reproductive strategies critical for their survival, proliferation, and effectiveness in sewage treatment systems. These strategies are finely tuned to wetland environments’ dynamic and often harsh conditions, enabling macrophytes to maintain robust populations and contribute significantly to treating wetlands’ ecological and functional stability.

One of the primary reproductive strategies aquatic macrophytes employ is vegetative reproduction, which allows for rapid colonization and expansion within a treatment system. Many species, such as *Typha* spp. and *P. australis*, propagate through rhizomes or stolons, horizontal structures that grow beneath or on the soil surface [112,113]. This form of asexual reproduction is particularly advantageous in stable, nutrient-rich environments such as constructed wetlands, where space and resources are readily available. *P. australis* demonstrates remarkable regeneration ability from rhizome and culm fragments, contributing to its invasive success [112]. These species can produce a significant biomass (10–30 t dry matter ha^−1^ y^−1^) and effectively remove nutrients from water [114,115], increasing blue carbon stocks and soil volume in marshes and potentially enhancing ecosystem resilience [116]. Their clonal growth strategies allow them to exploit heterogeneous resources effectively [117]. The ability to spread vegetatively ensures that these plants can quickly dominate a treatment area, forming dense stands that are highly effective in filtering pollutants, stabilizing sediments, and enhancing nutrient cycling. In addition to vegetative reproduction, many macrophytes also reproduce sexually, producing seeds that can be dispersed over long distances by water, wind, or animals [118]. Sexual reproduction is essential for maintaining genetic diversity within macrophyte populations, which is crucial for adapting to changing environmental conditions and resisting diseases and pests. For instance, seeds produced by species such as *Nymphaea* spp. and *Carex* spp. can remain dormant for extended periods, allowing them to germinate when conditions become favorable. This strategy ensures the persistence of macrophyte populations, even in fluctuating and unpredictable wetland environments.

The dual approach of both sexual and asexual reproduction gives macrophytes a significant ecological advantage. Vegetative reproduction enables rapid population expansion and immediate functional benefits within a wetland system, while sexual reproduction provides the genetic diversity necessary for long-term resilience and adaptability. This balance between the two reproductive modes allows macrophytes to maintain stable populations that can withstand the stresses of sewage treatment environments, such as varying water levels, nutrient loads, and contaminant concentrations. The population dynamics of macrophytes in sewage treatment systems are also influenced by environmental factors such as hydrology, nutrient availability, and competition with other plant species. High nutrient loads typical of sewage effluents often favor fast-growing species that can outcompete others, leading to monocultures or the dominance of particular species, such as *E. crassipes* or *L. minor*. While these dominant species can be highly effective in pollutant removal, their unchecked growth may require management interventions to prevent the clogging of water channels and maintain biodiversity within the system. Moreover, the reproductive success of macrophytes in these systems can be impacted by the presence of pollutants, such as heavy metals and organic contaminants, which may affect seed germination rates, vegetative propagation, and overall plant health [119,120]. However, many macrophytes have evolved tolerance mechanisms, such as enhanced antioxidant enzyme activity and efficient detoxification pathways [102], that allow them to reproduce successfully, even under contaminated conditions.

In summary, aquatic macrophytes’ reproductive strategies and population dynamics are critical factors in their ability to thrive in sewage treatment systems. By balancing rapid vegetative expansion with the genetic diversity provided by sexual reproduction, these plants ensure their long-term survival and effectiveness in enhancing the ecological functions of wetland treatment systems. Understanding these dynamics is crucial for optimizing the design and management of treatment wetlands, ensuring that macrophyte populations remain healthy, diverse, and capable of sustaining high levels of pollutant removal over time.

## 4. Selection Criteria and Examples of Macrophyte Species for Wetland-Based Treatment Systems

**Figure 2:**
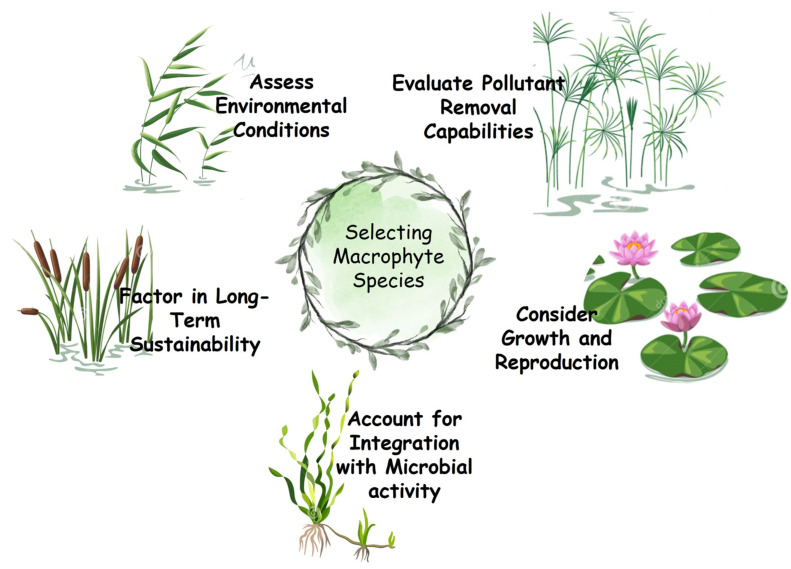
Steps for selecting macrophyte species for wetland-based treatment systems.

Selecting the appropriate macrophyte species for wetland-based treatment systems is a critical process that requires careful consideration of several environmental, biological, and functional factors. This process ensures that the chosen species not only thrives in the treatment environment but also optimizes the system’s efficiency in removing pollutants and maintaining ecological balance (Figure 2).

The first step in selecting macrophytes is to assess the environmental conditions of the treatment site. Key factors include water depth, soil composition, nutrient levels, and environmental stressors, such as pH, salinity, and temperature. The chosen species must demonstrate adaptability to these specific conditions [4]. Species with high phenotypic plasticity—those capable of altering their growth patterns, morphology, and physiology in response to environmental changes—are precious. For example, the species *P. australis* can thrive in various water depths and soil types, making it a versatile option for various wetland environments.

Next, the pollutant removal capabilities of potential macrophyte species must be evaluated [20,121]. This step involves identifying the primary contaminants in the wastewater, such as nutrients, heavy metals, or organic pollutants, and selecting species known for their efficiency in absorbing, accumulating, or transforming these substances. Next, the pollutant removal capabilities of potential macrophyte species must be evaluated [4,20]. This step involves identifying the primary contaminants in the wastewater, such as nutrients, heavy metals, or organic pollutants, and selecting species known for their efficiency in absorbing, accumulating, or transforming these substances. To begin, a comprehensive literature review should be conducted using scientific databases to identify species with documented efficacy in removing specific contaminants (Table 4). However, if the literature is lacking or species with proven tolerance are not well-documented, related species within the same genus can be considered due to their likely similar traits. In cases where information is scarce, experimental evaluation of candidate species can be undertaken, testing their performance under controlled conditions that mimic the treatment environment. Collaboration with research institutions and botanical gardens or leveraging unpublished data can also provide insights into lesser-known species with potential. Moreover, using functional traits as indicators, such as root morphology and biomass production, can guide the selection of species likely to perform well in pollutant removal. In some cases, genetic and biotechnological approaches may be explored to enhance the contaminant tolerance of certain species. If no adequate species are identified, field trials and adaptive management practices can be implemented to continuously refine species selection and optimize the treatment system’s effectiveness.

Another crucial consideration is the growth and reproduction characteristics of the macrophytes. Species exhibiting rapid growth and quickly establishing themselves within the treatment area are desirable, as they provide immediate benefits in pollutant removal and habitat stabilization. Additionally, species that reproduce vegetatively through structures such as rhizomes or stolons are particularly advantageous, as they can rapidly colonize new areas, ensuring continuous coverage and functionality of the treatment system [112,113]. However, maintaining genetic diversity through sexual reproduction is essential for long-term adaptability and resilience.

Integrating macrophytes with microbial activity in the treatment system is another vital factor. The root zones of macrophytes create microenvironments that support diverse and active microbial communities, which are essential for nitrification and denitrification [87]. Selecting species that enhance these microbial processes can significantly improve the overall efficiency of the treatment system. For example, *P. australis* contributes to nutrient uptake and fosters a microbial environment that degrades organic pollutants [87]. Finally, considering the chosen species’ long-term sustainability within the treatment system is essential. This involves selecting species resistant to local pests and diseases, requiring minimal maintenance, and contributing to the system’s long-term stability. Incorporating a diversity of species with complementary roles can enhance the resilience of the treatment system, ensuring that it can continue to function effectively under varying environmental conditions and potential disturbances.

In summary, selecting macrophyte species for wetland-based treatment systems should systematically consider environmental adaptability, pollutant removal capabilities, growth and reproduction characteristics, integration with microbial activity, and long-term sustainability. By carefully evaluating these factors, practitioners can optimize the design and management of treatment wetlands, ensuring their effectiveness and durability in treating wastewater and protecting water resources.

## 5. Challenges

While using aquatic macrophytes in wetland-based sewage treatment systems offers numerous benefits, several challenges must be addressed to harness their potential fully. One of the primary difficulties lies in the variability of environmental conditions, such as fluctuations in water levels, temperature, pH, and salinity [16]. Although macrophytes are generally adaptable, these environmental changes can cause stress and reduce efficiency. For example, sudden changes in water levels can disrupt root anchorage and hinder nutrient uptake, while extreme temperatures may impair photosynthesis and overall plant growth. Another significant challenge is the presence of complex contaminant mixtures in WWTP effluents. These mixtures often include nutrients, heavy metals, pharmaceuticals, and organic pollutants. While macrophytes can remove many of these substances, the interactions between multiple contaminants complicate treatment [4,5,143]. Some pollutants may inhibit the uptake or degradation of others, while others may accumulate to toxic levels, adversely affecting plant health and overall system performance.

The invasive potential of certain macrophyte species presents a significant challenge in wetland-based sewage treatment systems. Species such as *E. crassipes*, known for its rapid growth, can become invasive if not properly managed. While they are highly effective in pollutant removal, their uncontrolled proliferation can lead to clogged waterways, reduced oxygen levels, and negative impacts on local biodiversity [144]. While native and alien species can become invasive, alien species are particularly problematic when they disrupt local ecosystems, outcompeting native flora and altering the balance of biodiversity [145]. This makes it crucial to carefully select and manage species in wetland-based sewage treatment systems to avoid ecological harm. The success of these invasions is influenced by various factors, including the species’ growth and reproduction traits, environmental conditions, and the richness of native species in the area [144]. Management strategies have evolved, with biological control methods showing success, particularly against floating macrophytes in regions such as South Africa [146]. However, this has led to a shift in focus towards managing submerged and emergent invasive species, which can be equally problematic. Researchers now emphasize the need for holistic approaches to managing invasive macrophytes, considering their ecological impacts and potential benefits, such as their role in phytoremediation [18]. Balancing the effective use of these plants in treatment systems with the prevention of their invasive behavior is a complex issue that demands careful management.

Another critical challenge in macrophyte-based treatment systems is biomass management and disposal. As these plants grow and absorb contaminants, they must be periodically harvested to maintain system efficiency. However, disposing of this biomass, mainly when it contains high levels of absorbed pollutants such as heavy metals, presents a significant dilemma [3]. Developing safe and sustainable disposal methods is essential for preventing the reintroduction of contaminants into the environment, ensuring that the benefits of macrophyte-based treatment systems are not offset by environmental risks associated with biomass disposal.

Seasonal variation significantly impacts the performance of macrophyte-based systems. Many macrophytes enter a dormant phase during colder months, reducing growth rates and lowering pollutant removal efficiency [147,148,149]. This seasonality necessitates careful planning and management to ensure year-round effectiveness, potentially requiring supplementary treatment methods during periods of low performance. Seasonal changes in macrophyte biomass affect channel hydraulics, nutrient uptake, and water temperature patterns [150]. Additionally, macrophyte growth and senescence influence biofilm abundance, whole-stream metabolism, and nutrient cycling, which are crucial for maintaining the ecological functions of treatment systems throughout the year [151].

Despite the growing body of research on macrophyte-based treatment systems, significant knowledge gaps remain, particularly regarding the long-term effects of using certain species, their interactions with complex contaminant mixtures, and their response to climate change [152]. Addressing these challenges through continued research, adaptive management, and innovative practices is crucial to optimizing the use of macrophytes in wastewater treatment systems.

## 6. Practical Implications

Understanding the morphophysiological adaptations of aquatic macrophytes offers significant opportunities to optimize wetland-based treatment systems. These insights are crucial for refining system design and management practices, particularly in selecting macrophyte species that excel in nutrient absorption and pollutant removal. Strategic selection of species such as *P. australis* and *Typha* spp., known for their high efficiency in nutrient uptake and heavy metal sequestration, can enhance the overall performance of treatment systems. These species are particularly effective in environments with high nutrient loads and heavy metal contamination, ensuring the systems operate at their highest potential.

The adaptability of macrophytes to varying environmental conditions and contaminant types also supports dynamic and responsive system management. Continuous monitoring of plant health and system performance allows for informed adjustments, such as modifying planting densities or rotating species, to sustain optimal treatment efficiency. This adaptive management is essential for addressing fluctuations in effluent characteristics and environmental changes over time. Economic considerations are also vital in evaluating the use of macrophytes in treatment systems. Initial setup costs, ongoing maintenance expenses, and long-term benefits, such as improved water quality and reduced reliance on chemical treatments, must be considered. Financial incentives or subsidies can also support the broader adoption of macrophyte-based systems. Compliance with regulatory standards for wastewater treatment and environmental protection is crucial for successful implementation. Integrating these requirements into system design ensures a smoother operation and helps advocate for policies that support nature-based solutions, such as wetland restoration and green infrastructure. Public awareness and stakeholder engagement are crucial for successfully adopting and managing macrophyte-based systems. Educational programs and community outreach can help highlight these systems’ environmental and economic benefits, fostering support and participation from local governments, environmental organizations, and industry representatives.

## 7. Addressing Key Questions of the Review

### 7.1. How Do Morphophysiological Adaptations of Macrophytes Contribute to Their Survival and Efficiency in Sewage Treatment Systems?

The review has shown that adaptations such as aerenchyma formation, flexible leaf and stem structures, and specialized root systems enable macrophytes to thrive in nutrient-rich, waterlogged environments. These adaptations allow for efficient nutrient uptake, pollutant degradation, and sediment stabilization, which are crucial for the success of sewage treatment systems.

### 7.2. How Can These Adaptations Optimize the Design and Maintenance of Wetland-Based Treatment Systems?

By selecting species with specific adaptive traits, practitioners can design systems that are more resilient to environmental stressors and capable of maintaining high performance over time. This reduces the need for frequent maintenance and allows the systems to operate sustainably under various environmental conditions.

### 7.3. What Is the Significance of These Adaptations in Managing the Complex Composition of WWTP Effluents?

The ability of macrophytes to tolerate and process multiple contaminants simultaneously is critical for managing the complex composition of WWTP effluents. These adaptations enable plants to effectively remove a wide range of pollutants, ensuring that treatment systems remain effective even when faced with diverse and interacting contaminants.

### 7.4. How Do These Findings Influence Environmental Management and Policy?

The insights from this review support integrating nature-based solutions, such as macrophyte-based treatment systems, into environmental management and policy frameworks. This approach promotes sustainable water management practices and enhances the ecological and economic value of wastewater treatment systems.

### 7.5. What Are the Broader Ecological Benefits of Using Macrophytes in Wetland-Based Treatment Systems?

Beyond their role in pollutant removal, macrophytes contribute to broader ecosystem services, including habitat creation, carbon sequestration, and the maintenance of biodiversity. These benefits enhance wetland-based treatment systems’ overall ecological function and resilience, making them valuable components of environmental conservation efforts.

## 8. Conclusions and Future Perspectives

This study demonstrates that aquatic macrophytes exhibit significant morphophysiological adaptations that enable them to thrive in wetland-based sewage treatment systems, even under challenging environmental conditions. These adaptations, including structural features and functional mechanisms such as pollutant bioaccumulation and biofilm formation, allow macrophytes to effectively remove contaminants and contribute to the overall efficiency of treatment systems. Contrary to common misconceptions, macrophytes are not hindered by the conditions in sewage treatment systems but instead adapt and perform efficiently, enhancing the system’s resilience and effectiveness.

Future research should focus on exploring the specific adaptive traits of different macrophyte species to optimize their application in nature-based solutions for sewage treatment. Further studies should investigate how these adaptations can inform the design, implementation, and management of more efficient and resilient wetland-based systems. Additionally, optimizing environmental conditions for macrophyte growth and pollutant removal will be crucial in maximizing the benefits of these systems. Integrating macrophytes with complementary technologies, such as mechanical filters or chemical treatments, could provide more effective and sustainable solutions for wastewater management. This integrated approach would enhance the pollutant removal capacity and support environmental recovery efforts. The continuous improvement of sewage treatment practices, guided by a deeper understanding of macrophyte adaptations, can promote more sustainable water resource management and encourage the widespread adoption of nature-based solutions.

## Figures and Tables

**Table 1:** Aquatic macrophyte morphotypes and their functions in water treatment.

Macrophyte Morphotype	Characteristics	Function	Benefits to Treatment	Examples
Submerged	Completely submerged; leaves below the water surface	Absorbs nutrients directly from the water; provides oxygenation	Improves water quality; reduces turbidity; provides shelter for aquatic organisms	*Elodea canadensis*, *Vallisneria* spp.
Emergent	Roots submerged; vegetative parts extend above the water surface	Provides structural support; stabilizes substrate; creates habitats for wildlife	Contributes to water filtration and nutrient removal; offers diverse habitats	*Phragmites australis* (common reed), *Typha* spp. (cattail)
Free-Floating	Entirely float on the water surface, including leaves and roots	Absorbs nutrients directly from the water	Reduces nutrient loads; controls algal proliferation	*Eichhornia crassipes* (water hyacinth), *Lemna minor* (duckweed)
Floating-leaved	Leaves float on the water surface; the plant is anchored to the substrate	Captures sunlight for photosynthesis; submerged parts absorb nutrients	Provides shade to reduce algae growth; stabilizes substrate	*Nymphaea* spp. (water lilies), *Nelumbo nucifera* (lotus)

**Table 4:** Criteria for pollutant removal and macrophyte examples.

Criteria	Pollutant Removal Mechanisms	Species Examples and References
Nutrient Absorption and Accumulation	Efficient absorption of nutrients, such as nitrogen and phosphorus, through extensive root systems and adapted leaves.	*Typha latifolia* [66,122]
		*Phragmites australis* [123,124]
		*Cyperus papyrus* [125,126]
		*Eichhornia crassipes* [127,128]
Organic Pollutant Degradation	Ability to degrade organic pollutants through metabolic processes and interactions with microorganisms	*P. australis* [129,130]
		*Elodea canadensis* [131]
		*E. crassipes* [132,133]
		*Lemna* sp. [5,59,101]
		*Salvinia molesta* [60,74]
		*Typha* spp. [134]
Inorganic Pollutant Transformation and Stabilization	Sequestration and transformation of inorganic contaminants, such as heavy metals, into less toxic forms	*E. crassipes* [128]
		*Salvinia* spp. [135]
		*T. latifolia* [136]
Support for Microbial Activity	Creation of favorable conditions for microbial activity, which contributes to pollutant degradation and nutrient cycling	*T. latifolia* and *Thelypteris palustris* [137]
		*E. crassipes* [136,138]
		*P. australis* [139,140]
Heavy Metal Removal	Ability to remove and stabilize heavy metals through sequestration in plant tissues or chemical transformation	*E. crassipes* [128]
		*Salvinia* spp. [141,142]
